# Psychological Responses of the Patients in Cabin Hospital to the COVID-19 Outbreak: A Comparative Epidemiologic Analysis

**DOI:** 10.3389/fpsyg.2021.641167

**Published:** 2021-07-12

**Authors:** Yahui Wang, Mengyue Zhang, Qin Yin, Yincheng Wang, Pengcheng Yang, Chao Hu, Guogang Xu, Daoweng Wang, Xianzhi Li, Jibo He, Qinyong Hu, Xingguang Luo, Honggang Ren

**Affiliations:** ^1^Department of Psychology, Tsinghua University, Beijing, China; ^2^Silver School of Social Work, New York University, New York, NY, United States; ^3^Department of Respiratory and Critical Care Medicine, Hubei Provincial Hospital of Integrated Chinese and Western Medicine, Wuhan, China; ^4^Cancer Center, Renmin Hospital of Wuhan University, Wuhan, China; ^5^Department of Geriatric Medicine, The Eighth Medical Center of the Chinese PLA General Hospital, Beijing, China; ^6^The Second Medical Center & National Clinical Research Center for Geriatric Diseases, Chinese PLA General Hospital, Beijing, China; ^7^Department of Internal Medicine, Tongji Medical College, Huazhong University of Science and Technology, Wuhan, China; ^8^Department of Automation, Beijing University of Technology, Beijing, China; ^9^Beijing Huilongguan Hospital, Beijing, China; ^10^Department of Psychiatry, Yale University School of Medicine, New Haven, CT, United States; ^11^Department of Internal Medicine, Tongji Medical College, Huazhong University of Science and Technology, Wuhan, China

**Keywords:** COVID-19, cabin hospital, psychological responses, psychological assistance, depression, anxiety, resilience

## Abstract

The building of cabin hospitals in Wuhan has been proven to be clinically successful in curing mild-symptom COVID-19 patients shortly after the outbreak of COVID-19 in late 2019. At the same time, the psychological effect of patients being treated in cabin hospitals and the features of the psychological status of the whole society remained ambiguous. This study adopted a self-administrated questionnaire to investigate the stress, depression, and anxiety status of patients in cabin hospitals (*n* = 212) and healthy participants outside of Hubei province (*n* = 221) in a population level from February 29 to March 01, 2020. The research measured participants’ stress response, depression level, and anxiety level as well as their social support system and their resilience level. Results indicated that in this sudden outbreak of an unknown pandemic, all people (whether or not infected) showed a generally high level of stress, depression, and anxiety, regardless of age, gender, education level, and employment. It also showed that people with a lower level of psychological resilience and social support reported more severe symptoms of depression, anxiety, and stress. Moreover, the research also found a positive effect of cabin hospitals on the psychological recovery of COVID-19 patients. Stress response of patients increased after entering into cabin hospitals, while after 3–4 weeks’ treatment, patients showed a decrease in their depression and anxiety levels. This research advances the understanding of COVID-19 and gives suggestions to optimize the design and the allocation of resources in cabin hospitals and better deal with the unknown pandemics in the future.

## Introduction

Beginning in December 2019, the coronavirus disease 2019 (COVID-19) has swept over 200 countries and caused 106,508,151 cases including 2,323,815 deaths (data collected on February 08, 2021^1^). Symptoms of COVID-19 usually include fever, cough, upper airway congestions, fatigue, dyspnea, and headache (Wang et al., 2020). It has been proven that people without any symptom could also be COVID-19 positive and spread the virus. Shortly following the rapid spread of the coronavirus, a number of studies have been published around the genomic characterization of COVID-19 to support clinical treatment (Brooks et al., 2020; Sun et al., 2020; Wu and McGoogan, 2020).

Cabin hospitals were developed for the first time in China to tackle the COVID-19 outbreak (Chen et al., 2020). In addition to providing medical treatment, cabin hospitals also offered psychological and physical interventions such as daily exercise, recovery diaries, emotional painting, and letter writing to help patients’ recovery and thus better thrive during a disruptive period in patients’ lives (Shu et al., 2020). In research from Zhang et al. (2020), they recruited 296 patients from Cabin Hospital in Wuhan, Hubei, China, with mild symptoms of COVID-19. This study shows that after taking the general demographics into consideration, higher levels of resilience were associated with lower anxiety and depression among mild COVID-19 patients in Wuhan, China. In research from Guo et al. (2020), they investigated and compared the mental status of hospitalized patients with mild physical symptoms and matched controls that were COVID-19 negative. The results indicate that significant psychological distress was experienced by hospitalized COVID-19 patients and that levels of depressive features may be related to the inflammation markers in these patients. Qualitative analysis also revealed similar results with respect to patient reports of negative feelings, including fear, guilt, and helplessness. Stigma and uncertainty of viral disease progression were two main concerns expressed by COVID-19 patients.

Previous study shows that high risk of mental health problems is associated with COVID-19 (Ma et al., 2020). Depression is highly prevalent in clinically stable patients with COVID-19 (Lei et al., 2020). Recent research also shows that both patients and their relatives suffer from high levels of anxiety and related pandemic worries, with lower levels of depressive symptoms. While increased anxiety among patients was associated with feelings of isolation, increased anxiety among relatives was associated with a feeling of not being protected by the hospital (Dorman-Ilan et al., 2020).

To add in and further explore the psychological impact of COVID-19, we aimed to measure participants’ stress, depression, and anxiety responses to COVID-19, including stress, depression, and anxiety level. We also measured participants’ social support and resilience level to examine the hypothesis that under the threat of deadly contagions, people with a high level of resilience are less likely to develop negative psychological symptoms, and proper social supports can improve patients’ resilience and assist their holistic recovery.

## Materials and Methods

### Study Design and Participants

The study conducted a cross-sectional research. It measured the stress, depression, and anxiety responses among COVID-19 patients in Wuhan cabin hospitals. To better illustrate the psychological effects of cabin hospital, the study also introduced a group of healthy participants outside of Hubei province in a population level as control group.

The study adopted an anonymous self-report questionnaire via WeChat-based survey program Questionnaire Star with items (taking around 20 min), and the data were collected between February 29 and March 1, 2020, which was the outbreak period of COVID-19 in Wuhan. Participants were recruited through convenience sampling. Following the principle of not disturbing patient’s rest, researchers explained the purpose of the study to them. Then, researchers introduced the contents of the questionnaire and explained how to complete it. Lastly, they distributed a Questionnaire Star link (an online crowd sourcing platform in China) to the electronic questionnaire by scanning a Quick Response code. A total of 433 participants responded, and all of them were included in the data analysis of this study. The sample was composed of 212 COVID-19 patients living in different Wuhan cabin hospitals, and a control group of 221 participants from other districts in China without a COVID-19 diagnosis.

This study was approved by the Ethical Committee of the Union Hospital, Tongji Medical College, Huazhong University of Science and Technology. Signed informed consent was obtained online from all participants.

### Survey Questionnaire

The questionnaire contained basic geographic and demographic information, including gender, age, education, occupation, and the date they were hospitalized (only for Cabin patients). The study also utilized five standardized scales, including Stress Response Questionnaire (SRQ), Patient Health Questionnaire (PHQ-9), Generalized Anxiety Disorder (GAD-7), Connor – Davidson Resilience Scale (CD – RISC), and Social Support Rate Scale (SSRS).

#### Stress Response Questionnaire (SRQ)

Stress Response Questionnaire is a 5-score Likert scale and used to measure stress response through 28 items. The total score of SRQ is 140, and the higher score indicates higher levels of stress response. The Chinese version (Cronbach’s alpha = 0.964) adopted by this study had been proven to be valid and reliable (Zhong et al., 2004; Crum et al., 2013).

#### Patient Health Questionnaire (PHQ-9)

Patient Health Questionnaire is a nine-item scale and considered as a brief diagnostic and screening instrument in non-psychiatric settings for depressive symptoms by a total score of 27, and the higher score indicates higher levels of depression response. The Chinese version (Cronbach’s alpha = 0.924) adopted by this study had been proven to be valid and reliable (Xiong et al., 2015).

#### Generalized Anxiety Disorder (GAD-7)

Generalized Anxiety Disorder and its Chinese version (Cronbach’s alpha = 0.952) are four-point scales which are treated as a reliable and valid measure of anxiety response in the general population (Löwe et al., 2008). The total score of GAD-7 is 21, and the higher score indicates a higher level of anxiety response.

#### Connor – Davidson Resilience Scale (CD-RISC)

Psychological resilience refers to an individual’s ability to thrive despite adversity. The patients’ resilience was measured by CD-RISC. The original version was developed by Connor and Davidson (2003) and was translated to Chinese by Yu and Zhang (2007), with authorization from the original developers. The CD-RISC in Chinese (Cronbach’s alpha = 0.890) consists of 25 items representing three dimensions: tenacity, strength, and optimism. It employs a five-point Likert-type scale (0 = “never” to 4 = “almost always”). The total scores range from 0 to 100, and higher total scores indicate higher levels of resilience (Connor and Davidson, 2003; Yu and Zhang, 2007). Since all participants responded to the religious items (item 3 and item 9) as “0 = never,” we excluded these two items in the following analysis.

#### Social Support Rate Scale (SSRS)

Social Support Rate Scale is a 10-item scale which was created in 1986 with three factors, including subjective support, objective support, and availability (McLean, 1992). The total score of SSRS is 66, and the higher score indicates higher levels of social support. It has been proved that its Chinese version (Cronbach’s alpha = 0.798) has high construct validity, good content validity, and high reliability for the application in China (Xiao, 1994).

### Data Analysis

Categorical variables were summarized as counts and percentages and were compared using Chi-square tests between the groups. Continuous variables were described as mean and standard error and were compared using *t*-test analysis or MANCOVA. According to the score of psychological resilience, all participants were divided into two groups by the median of their scores, i.e., the lower psychological resilience group and the higher psychological resilience group. In addition, all participants were divided into the higher social support group and the lower social support group based on the median of their social support score. General information analyses of the sample data (sociodemographic characteristics, general stress, general stress, depression, and anxiety responses) were performed using SPSS (Version 23, IBM); moderation analysis and the analysis of psychological recovery of COVID-19 patients on a small-time scale were conducted using SAS (Version 9.4, The SAS Institute, Cary, NC, United States). A two-sided *p*-value < 0.05 was considered statistically significant. Partial eta squared ($\eta_{p}^{2}$) was used as the measure of effect size, and 0.01, 0.06, and 0.14 represented the small, medium, and large effect sizes, respectively (Cohen, 1973, 1988).

## Results

### Demographic Profile

Participants consisted of 198 men and 235 women in total. The majority of them were between the ages of 18 and 60 and had undergraduate degree or below, as shown in Table 1.

**TABLE 1:** General information of the sample data.

**Sociodemographic factors**	**Cabin patients**	**Control group**	**Test value**	***p***
	***n* = 212 n (%)**	***n* = 221 n (%)**		
**Sex**			*U* = 90713	0.171
Men	100 (47.2)	98 (44.3)		
Women	112 (52.8)	123 (55.7)		
**Age group (years)**			*χ2* = 195	0.003**
18–35	70 (33.0)	112 (50.7)		
36–60	131 (61.8)	100 (45.2)		
61 and above	11 (5.2)	9 (4.1)		
**Educational level**			*U* = 75464	< 0.001***
K12 and under	103 (48.6)	46 (20.8)		
Undergraduate	101 (47.6)	141 (63.8)		
Graduate	8 (3.8)	34 (15.4)		
**Employment status**			*χ2* = 164	0.188
Employed	94 (44.3)	105 (47.5)		
Unemployed	118 (55.7)	116 (52.5)		

U = Mann–Whitney U test. χ2 = chi square test. **p < 0.01, ***p < 0.001.

Among all the patients in cabin hospitals, 100 (47.2%) were men and 112 (52.8) were women. One hundred forty-two (67%) patients were over the age 36. There were 101 (47.6%) patients with undergraduate degrees, and 103 (48.6%) had K-12-level educations and under. Ninety-four (44.3%) were employed and 118 (55.7%) were unemployed. As for the control group, 98 (44.3%) were men and 123 (55.7%) were women. One hundred twelve (50.7%) cases were under the age 36. Most (63.8%) participants in the control group had undergraduate degrees. One hundred and sixteen (52.5%) of them were employed.

### General Stress, Depression, and Anxiety Responses

In order to investigate whether the psychological resilience or social support was a moderator between cabin and psychological responses (stress, depression, and anxiety), respectively, the moderation analysis (Bootstrap samples = 5,000) was used; results of these models are shown in Table 2. It indicated that for both psychological resilience and social support, no significant moderation effect was identified in the analysis.

**TABLE 2:** Regression analysis of psychological resilience or social support between cabin and stress, depression, and anxiety responses.

**Psychological responses**	**Stress**				**Dpression**				**Anxiety**			
****	**β**	***T***	***p***	**95%CI**	**β**	***t***	***p***	**95%CI**	**β**	***t***	***p***	**95%CI**
Psychological resilience	−0.04	−0.38	0.71	−0.22∼0.15	0.004	0.14	0.89	−0.05∼0.05	0.02	1.14	0.26	−0.02∼0.06
Social support	0.44	1.70	0.09	−0.07∼0.96	0.02	0.29	0.77	−0.11∼0.14	0.03	0.59	0.56	−0.07∼0.13

*Independent variable=cabinDependent variables=psychological responses (stress, depression, and anxiety).Moderators=psychological resilience and social support.*

**FIGURE 1:**
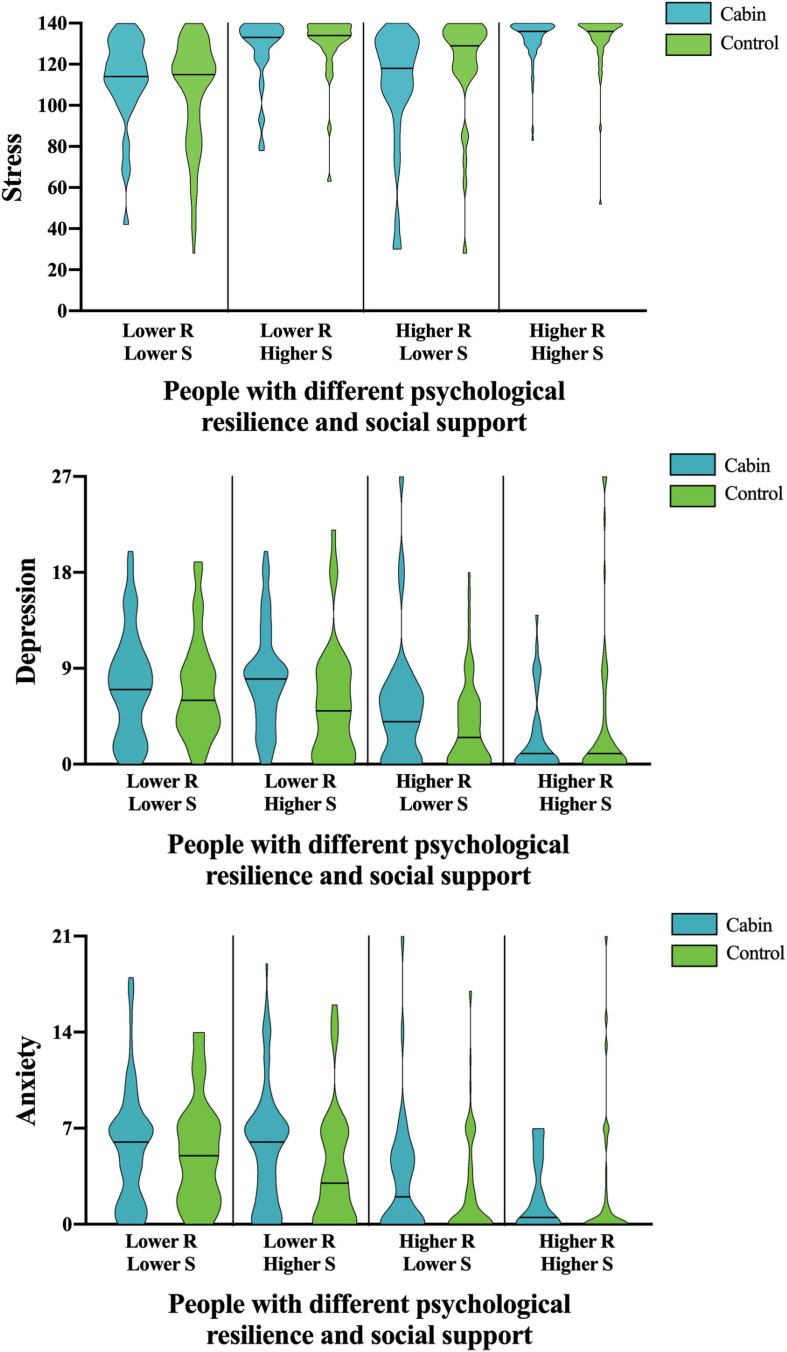
Psychological responses in different levels of psychological resilience and social support.

Therefore, using the score of stress, depression, and anxiety as dependent variables, a 2 (Cabin vs. Control) × 2 (High psychological resilience vs. Low psychological resilience) × 2 (High social support vs. Low social support) MANCOVA was used to analyze the stress, depression, and anxiety level of participants. Recent research shows that for older adults who suffered Covid-19, their depression levels differed significantly in marital status, living situation, education level, household income, subjective health status, and infected cases of acquaintances (Liang et al., 2021). In this study, gender, age, and educational level were included as covariates, yet there were no significant effects in psychological response. Therefore, we conducted a more in-depth demographic data analysis on stress, depression, and anxiety to explore whether these responses were common among human beings in the outbreak of the COVID-19 epidemic, regardless of gender, education level, age, and employment status. As shown in Table 3 and Figure 1, the interaction among these three independent variables was significant in stress and depression. All the two-way interactions were not significant. The main effect was analyzed as following.

**TABLE 3:** Analysis of variance of stress, depression, and anxiety responses under different conditions.

**Variables**	**Stress**			**Depression**			**Anxiety**		
	***F***	***p***		***F***	***p***		***F***	***p***	
Cabin	< 0.001	0.98	< 0.001	2.38	0.12	0.01	1.98	0.16	0.01
Social report	4.79	0.03*	0.01	1.76	0.19	0.004	1.62	0.20	0.004
Psychological resilience	70.91	< 0.001***	0.14	36.25	< 0.001***	0.08	32.61	< 0.001***	0.07
Cabin × social report	2.44	0.12	0.01	0.17	0.68	< 0.001	< 0.001	0.99	< 0.001
Social report × psychological resilience	0.29	0.59	0.001	0.81	0.37	0.002	0.81	0.37	0.002
Cabin × psychological resilience	< 0.001	0.99	< 0.001	0.85	0.36	0.002	0.04	0.84	< 0.001
Cabin × social report × psychological resilience	5.82	0.02*	0.01	4.80	0.03*	0.01	2.40	0.12	0.01
Gender	0.05	0.83	< 0.001	0.80	0.37	0.002	0.04	0.85	< 0.001
Age	0.52	0.47	0.001	2.41	0.12	0.01	1.51	0.22	0.004
Educational level	3.61	0.06	0.01	0.51	0.48	0.001	0.20	0.66	< 0.001

*p < 0.05, ***p < 0.001.

### Ubiquity of Stress, Depression, and Anxiety in and Outside of Cabin Hospital

As shown in Table 4, male participants had a higher stress level than females in both the cabin and control groups, while female participants reported more severe depressive and anxiety symptoms in the cabin group but lower in the control group. The young participants in cabin hospitals reported higher levels of stress and lower levels of depression and anxiety, while the results of the control group were the opposite. For the education level, participants with K12 and under reported the lowest stress in cabin and the highest depression and anxiety. Moreover, in the control group, the participants with a graduate degree reported the lowest depression, while the K12 and under participants reported the lowest stress and anxiety. Employed people in the cabin group reported higher stress and lower depression and anxiety than those in the control group. There was no significant difference in psychological response among the variables of sex, age, education, and employment status in the cabin group except the level of stress in education, and no significant difference was found in the control group except the level of depression and anxiety in age.

**TABLE 4:** Stress, depression, and anxiety responses based on sociodemographic characteristics.

**Variable**	**Stress**					**Depression**					**Anxiety**				
	**M**	**SD**	**p**	**t**	**d**	**M**	**SD**	**p**	**t**	**d**	**M**	**SD**	**p**	**t**	**d**
Cabin	121.16	21.45	0.400	0.842	0.081	5.79	5.04	0.715	0.367	0.035	4.11	4.35	0.650	0.454	0.044
Control group	119.29	24.51				5.60	5.77				3.91	4.55			
**Cabin sex**															
Men	123.43	20.47	0.145	1.463	0.201	6.140	5.81	0.344	0.933	0.131	4.12	4.84	0.971	0.036	0.005
Women	119.16	22.18				6.23	4.23				4.130	3.89			
**Cabin age group (years)**															
18–35	124.20	21.44	0.158	/	/	5.49	5.27	0.575	/	/	3.87	4.50	0.661	/	/
36–60	119.76	21.52				5.95	5.047				4.24	4.35			
61 and above	118.36	20.38				5.91	3.39				4.00	3.58			
**Cabin educational level**															
K12 and above	117.60	23.07	0.042*	/	/	5.92	4.83	0.819	/	/	4.32	4.26	0.945	/	/
Undergraduate	124.37	19.65				5.75	5.33				3.94	4.54			
Graduate	126.13	15.19				4.63	4.07				3.50	3.07			
**Cabin employment status**															
Employed	121.80	21.55	0.210	0.388	0.054	5.60	4.95	0.269	0.507	0.070	3.69	4.13	0.247	1.248	0.173
Unemployed	120.64	21.44				5.95	5.12				4.44	4.51			
**Control group sex**															
Men	120.83	24.67	0.407	0.831	0.113	6.13	6.00	0.292	1.056	0.143	4.05	4.52	0.217	1.239	0.168
Women	118.07	24.41				6.12	5.58				3.98	4.56			
**Control group age (years)**															
18–35	119.95	23.15	0.951	/	/	6.44	6.38	0.012*	/	/	4.44	4.95	0.024*	/	/
36–60	117.69	26.73				4.96	5.04				3.62	4.13			
61 and above	128.89	10.12				2.33	2.83				0.67	1.12			
**Control group educational level**															
K12 and above	115.00	28.36	0.225	/	/	5.52	5.59	0.757	/	/	3.54	4.42	0.639	/	/
Undergraduate	120.67	23.59				5.66	5.95				4.07	4.76			
Graduate	119.38	22.66				5.47	5.38				3.77	3.89			
**Control group employment status**															
Employed	113.91	27.85	0.134	3.120	0.427	6.43	6.15	0.505	2.042	0.275	4.46	4.74	0.228	1.695	0.590
Unemployed	124.16	19.93				4.85	5.31				3.42	4.33			

M = mean value. SD = standard deviation. *p < 0.05.

Among 433 participants, there was no significant difference in stress, depression, and anxiety responses between cabin and control groups, even though the general levels of stress, depression, and anxiety in cabin patients were higher than in the control group. For both cabin patients and control participants, their stress responses were at a relatively high level (total score = 140), as shown in Table 4. Therefore, when faced with COVID-19, there was a universal response in stress, depression, and anxiety among all participants, regardless of whether they have been infected or not.

### Advantages of Higher Psychological Resilience and Higher Social Support

The main effect of psychological resilience was significant in stress, depression, and anxiety. Figure 1 shows that people with higher psychological resilience presented lower depression and anxiety reactions, but a higher stress response than those with lower psychological resilience. As shown in Table 3 and Figure 1, the main effect of social support was only significant in stress, and it indicated that people with higher social support had higher stress than those with lower social support. No other significant effect was found.

Although people with higher psychological resilience and higher social support had better psychological status than those with lower psychological resilience and lower social support, the results were similar and common in the real world (Yu and Zhang, 2007). However, the ubiquity of stress, depression, and anxiety responses in and outside of cabin hospitals was greater than expected. Therefore, we conducted a more in-depth data analysis on a small-time scale.

### Psychological Recovery of COVID-19 Patients on a Small-Time Scale

**FIGURE 2:**
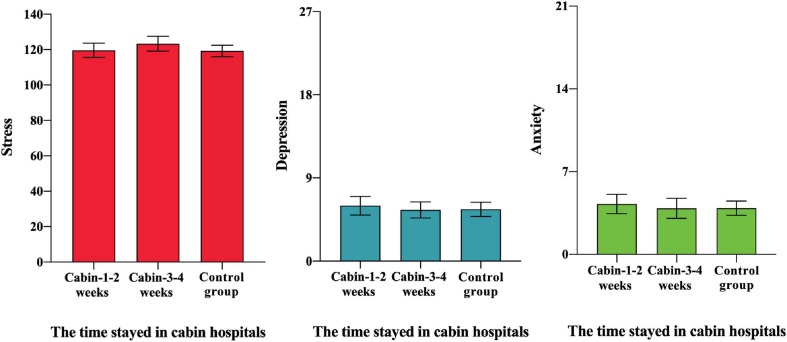
Psychological responses of cabin patients based on the time they stayed in cabin hospitals.

Patients were divided into two groups: patients who entered into the cabin hospital for 1–2 weeks (Cabin-1-2) and patients who came into the cabin hospital for 3–4 weeks (Cabin-3-4). Then, we compared the Cabin-1-2 group, Cabin-3-4 group, and Control group from three aspects: stress, depression, and anxiety. Even though there was no significant difference in the general levels of stress, depression, and anxiety among the Cabin-1-2 group, Cabin-3-4 group, and Control group, we discovered a positive effect on psychological recovery of COVID-19 patients on a time scale. Comparing the mean in depression and anxiety, the result shows that Cabin-1-2 group > Control group > Cabin-3-4 group, and in stress, Cabin-3-4 group > Cabin-1-2 group > Control group. Patients reported a higher level of anxiety and depression when they first entered cabin hospitals, but after receiving proper medical treatment and variable psychological support such as one-to-one remote psychological assistance, psychological crisis intervention, breathing relaxation techniques, meditation, and acupoint massage from medical staff, volunteers, and other patients in cabin hospital, patients reported improvement in their psychological status in 3–4 weeks, as shown in Table 5 and Figure 2. The levels of anxiety and depression were even lower than those of control people (not infected with COVID-19). At the same time, they suffered higher stress responses. Psychological recovery is an essential part of holistic recovery. It indicated that the cabin hospital could treat COVID-19 patients not only medically but also psychologically.

**TABLE 5:** Stress, depression, and anxiety responses of cabin patients based on the time they stayed in cabin hospitals.

**Variable**	**Stress**			**Depression**		**Anxiety**			
	***M***	***SD***	***p***	***M***	***SD***	***p***	***M***	***SD***	***p***
**Cabin-1-2**	119.57	22.46	0.358	5.99	5.61	0.771	4.26	4.55	0.760
**Cabin-3-4**	123.30	19.92		5.52	4.16		3.90	4.57	
**Control group**	119.29	24.51		5.60	5.77		3.91	4.05	

*M = mean value. SD = standard deviation.*

## Discussion

### Findings

The outbreak of COVID-19 has not only posed a threat to people’s lives and physical health but also caused tremendous stress, depression, and anxiety responses and affected the mental health of people (Torales et al., 2020). Past experiences have shown that the mental health implications have greater prevalence and economical and social impacts than the pandemic itself (Huang and Zhao, 2020; Torales et al., 2020). The main findings of the research demonstrated that there was a universally high level of stress, depression, and anxiety among people, regardless of their age, gender, occupation, and education level and whether they have been infected with COVID-19. There was no significant difference in psychological response between cabin and control participants when faced with COVID-19, which means that the psychological counseling, targeted humanistic care, and professional mental health services in cabin hospitals are critical for patients to reduce the psychological harm caused by the pandemic and recover to the same level as healthy people.

An interesting finding in the research is about the relationship between education level and stress response of COVID-19 patients in cabin hospitals. In the healthy control group, the lower education level participants (K12 and under) had lower stress than the higher education level participants (Undergraduate and Graduate). Meanwhile, the data of patients in cabin hospitals reflect significant differences in stress level regarding education levels: the higher education level participants had higher stress than the lower education level participants. Sylvie Briand, director of Infectious Hazards Management at WHO’s Health Emergencies Program, believed that infodemic risks, which is a global epidemic of misinformation, spread rapidly through social media platforms and other outlets and pose a serious problem for public health. A potential explanation is that people with higher education or having better ability to obtain information is more likely to be exposed to the spreading panic in social media. Such results were generally consistent with the previous research suggesting that the more educated and younger people experienced a relatively higher level of anxiety and stress (Mahoney et al., 2015; Liu et al., 2020). This may be ascribed to the fact that the educated people receive more information about COVID-19 through different media than people who are less educated, so they are more likely to know the severity of contracting COVID-19. Therefore, younger and more educated people who have more knowledge about COVID-19 are more likely to feel stress. Intensive exposure to the mass of information related to COVID-19 rapidly consumed their psychological resources which then generated stress responses.

### Limitations and Future Directions

This study has several limitations, one of which was the data we used. The data of COVID-19 patients was only collected in Wuhan cabin hospitals, and we have a notable deficit of participants from cabin hospitals in other provinces outside Hubei, which may cause selection bias. Second, as participants were included in our study on a voluntary basis, there may be a response bias among the volunteers, and the result lacks clinical evaluation. Third, the data sampling of the control group was voluntary and conducted using self-administered scales or questionnaires, so the representativeness and external validity cannot be guaranteed.

During the outbreak, the general public and patients around the world are under insurmountable psychological pressure and burdened with various psychological problems, such as anxiety, fear, depression, and stress (Sun et al., 2020; Yang et al., 2020). In many countries, there is a shortage of medical supplies. The public and patients even suffered substantial psychological trauma when facing death (Bridgland et al., 2021). In the outbreak of COVID-19, mental health problems are often behind the consideration of physical health (Zhou et al., 2020); as a result, a large number of patients with psychological diseases will appear after the end of the epidemic (German and Latkin, 2011). In addition, even though the cabin hospital provided the remote psychological counseling and psychological crisis intervention for patients to alleviate anxiety and panic, it is merely a temporary mobile medical space (Shu et al., 2020), which means that the psychological intervention of the patient will be interrupted after the patients discharged from the hospital.

The study reflects a need to construct a long-term mechanism for public emergent mental health support systems during the epidemic outbreak. It shows that the current reserve resources, coverage, management system, social support, collective psychological education, and the effectiveness of psychological assistance are not satisfactory for public mental health, and all of them need to be optimized, from the result of the psychological response of the control group. Therefore, the psychological resources should be integrated and thus contribute to the construction and optimization of the long-term emergency psychological assistance system in epidemics.

## Conclusion

In conclusion, facing COVID-19, an unknown severe epidemic to people, all people (whether infected or not) have suffered from general stress, depression, and anxious emotions, regardless of age, gender, education, and employment. This study explored the general psychological response of people during the outbreak of COVID-19 and found that the cabin hospital helped the patients’ psychological recovery to some extent. Cabin hospitals mainly treated mild-symptom patients who had a relatively weak psychological status in the early stage of entering into cabin hospitals. However, after 3–4 weeks of treatment, their mental state (except stress response) had been improved, and their depression and anxiety symptoms were lower than healthy people. Therefore, the cabin hospital might play a vital role in the relief of psychological stress, depression, and anxiety of patients in the spread of epidemics. Moreover, the lower the levels of psychological resilience and social support, the more negative these stress, depression, and anxiety responses. The findings were conducive to a better understanding of COVID-19 and encouraged further exploration of cabin hospitals. This research could promote the design and optimization of the allocation of resources of cabin hospitals and improve the construction of epidemic psychological counseling and defense systems, thus contributing to better deal with the outbreak of unknown epidemics in the future.

## Data Availability Statement

The raw data supporting the conclusions of this article will be made available by the authors, without undue reservation.

## Ethics Statement

The studies involving human participants were reviewed and approved by the Ethical Committee of the Union Hospital, Tongji Medical College, Huazhong University of Science and Technology. The patients/participants provided their written informed consent to participate in this study.

## Author Contributions

YHW, MZ, QY, YCW, JH, QH, XGL, and HR conceived and planned the study. PY, CH, GX, DW, and XZL carried out the study. YHW, MZ, QY, YCW, JH, QH, and XGL contributed to the interpretation of the results. YHW, MZ, QY, and YCW took the lead in writing the manuscript. All authors provided critical feedback and helped shape the research, analysis, and manuscript.

## Conflict of Interest

The authors declare that the research was conducted in the absence of any commercial or financial relationships that could be construed as a potential conflict of interest.

## Footnotes

1 https://www.worldometers.info/coronavirus
